# Emotion Classification Based on Pulsatile Images Extracted from Short Facial Videos via Deep Learning

**DOI:** 10.3390/s24082620

**Published:** 2024-04-19

**Authors:** Shlomi Talala, Shaul Shvimmer, Rotem Simhon, Michael Gilead, Yitzhak Yitzhaky

**Affiliations:** 1 Department of Electro-Optics and Photonics Engineering, School of Electrical and Computer Engineering, Ben-Gurion University of the Negev, Beer Sheva 84105, Israel; talala42@gmail.com (S.T.);; 2 School of Psychology, Tel Aviv University, Tel Aviv 39040, Israel

**Keywords:** emotion classification, remote emotion recognition, rPPG, camera-based PPG, pulsatile signal, deep learning

## Abstract

Most human emotion recognition methods largely depend on classifying stereotypical facial expressions that represent emotions. However, such facial expressions do not necessarily correspond to actual emotional states and may correspond to communicative intentions. In other cases, emotions are hidden, cannot be expressed, or may have lower arousal manifested by less pronounced facial expressions, as may occur during passive video viewing. This study improves an emotion classification approach developed in a previous study, which classifies emotions remotely without relying on stereotypical facial expressions or contact-based methods, using short facial video data. In this approach, we desire to remotely sense transdermal cardiovascular spatiotemporal facial patterns associated with different emotional states and analyze this data via machine learning. In this paper, we propose several improvements, which include a better remote heart rate estimation via a preliminary skin segmentation, improvement of the heartbeat peaks and troughs detection process, and obtaining a better emotion classification accuracy by employing an appropriate deep learning classifier using an RGB camera input only with data. We used the dataset obtained in the previous study, which contains facial videos of 110 participants who passively viewed 150 short videos that elicited the following five emotion types: amusement, disgust, fear, sexual arousal, and no emotion, while three cameras with different wavelength sensitivities (visible spectrum, near-infrared, and longwave infrared) recorded them simultaneously. From the short facial videos, we extracted unique high-resolution spatiotemporal, physiologically affected features and examined them as input features with different deep-learning approaches. An EfficientNet-B0 model type was able to classify participants’ emotional states with an overall average accuracy of 47.36% using a single input spatiotemporal feature map obtained from a regular RGB camera.

## 1. Introduction

Emotions are intricate, subjective experiences that arise from an individual’s physiological state, thoughts, and external stimuli. They play a crucial role in human life, influencing behavior, thoughts, actions, and social interactions. Extensive research has been conducted to explain the nature and functions of emotions and their significance for the field of human–computer interaction systems [1,2].

The most popular and widely studied approach for emotion classification is based on facial expressions [3,4]. Another popular feature used for emotion classification is voice [5]. Traditional approaches extract features from a facial image or a speech signal and then classify emotions based on feature characteristics. Recent deep learning (DL)-based approaches carry out the emotion recognition problem by integrating both feature extraction and classification processes into a single composite operating process. Methods based on facial expressions and speech signals demonstrate classification success rates surpassing 90% in classifying emotions [3,4,5]. These visual-audio techniques are based on contactless emotion detection and do not contain non-visible physiological characteristics. Nonetheless, facial expressions and voice may correspond to communicative intentions, or can potentially be manipulated to deceive, rather than authentically reflect, an individual’s genuine emotional state.

It has been established through various studies that the autonomic nervous system (ANS) plays a significant role in emotion regulation [6,7]. Operating within the peripheral nervous system, the ANS assumes control of a spectrum of involuntary bodily functions, spanning cardiac muscle contractions, visceral activities, and glandular operations. This intricate regulatory network extends its influence over critical physiological parameters, including heart rate (HR), blood pressure (BP), respiration rate, body temperature, perspiration, and galvanic skin response. These unconscious functions can be detected and monitored by different wearable sensors such as electromyography (EMG), electrocardiogram (ECG), and photoplethysmogram (PPG). In addition to the above, electroencephalogram (EEG) and functional magnetic resonance imaging (fMRI), which correspond to the central nervous system (CNS), are popular and important sensors for understanding the function of the human brain and are also studied for emotion recognition [8,9,10]. In contrast to facial expression/voice-based techniques, physiological signal responses may provide a more reliable representation of the actual emotional state and are harder to manipulate. However, their measurement methods, as stated above, are based on wearable/contact devices, which may be difficult to implement in everyday situations, especially for long-term monitoring, and can cause some discomfort or even impact the individual’s emotional state.

In light of this, and with the recent growth of mobile healthcare for daily healthcare or clinical environments, several attempts have been made to develop methods for detecting and monitoring physiological signals remotely. One of the most promising approaches, called camera-based PPG (cbPPG) or remote PPG (rPPG) [11,12], extracts the blood volume pulse (BVP) signals from a skin video recording (mainly from the face or from the hand). These methods measure the changes in blood volume through the amount of light transmitted or reflected after irradiating the skin region [13,14]. The extracted BVP signal also contains information related to cardiovascular activity and heart rate, as well as hemoglobin concentration [11,12,13,14,15,16].

Following that, in recent years, research has attempted to develop novel methods for the assessment of a person’s emotional state based on rPPG, mainly from visual cameras (VIS) [17,18,19,20,21]; this approach eliminates the need for uncomfortable sensors and offers the unique advantage of enabling spatial physiological measurement and visualization of the peripheral signals using only one sensor. However, the majority of studies in which PPG signals are extracted from facial video recordings involve averaging all signals from the entire face or specific predefined regions [18,19,20,21]. This conventional approach fails to account for the variability in signal characteristics across different facial regions, largely attributed to the complex influence of the ANS [22]. Consequently, this approach may inadvertently obscure crucial spatiotemporal variations in cardiovascular activities occurring across the face. 

Recognizing this limitation, which can be found in a recent work [17], we proposed a machine learning model to predict emotional states based on physiological changes across the face, which can be related to spatial rPPG methods. This research extracted transdermal spatiotemporal features based on the maximum and minimum values of the spatial rPPG signal obtained from three cameras, which included the visual spectrum range, the near-infrared (NIR) range, and the long-wave infrared (LWIR) thermal range. These features were used to train a CatBoost classifier, and the best overall average accuracy achieved was 44%. Additionally, the results produced spatial feature importance patterns associated with different emotional states. 

In the current study, we aim to improve, simplify, and validate the previous work [17]. As in [17], we pursued a “true” contactless (i.e., remotely) emotion classification from short (four seconds) multispectral facial videos of subjects, who passively viewed emotion-eliciting videos, and classified an individual’s emotional state based on features extracted from pulsatile images, which do not rely on stereotypical facial expressions. Validation of the ability to classify emotional states in our dataset was motivated by the fact that emotions in this dataset are hardly expressed. We improved the extraction and pre-processing of the physiological pulsatile-based signal used for classification purposes and examined various DL methods for classification using different input features. From the pulsatile images detected based on extracted heartbeat signals, we built high-resolution physiological features and fed them into suitable DL models for classifying the emotional state. 

The proposed method has several key advantages, with respect to the previous work of [17], and includes the following:

1. In this study, we used a DL-based classification model that achieved an overall accuracy of 47.36% using a single input feature map that can be obtained from a regular RGB camera, which has improved upon the previous work that achieved an overall accuracy of 44% that was trained with a block of seven input feature maps obtained from multispectral signals that also included the thermal and the NIR cameras. This means a better applicability in terms of the cost and complexity of the setup.
2. Unlike the previous work, here we achieve an estimated heart rate by first performing skin region segmentation, which effectively focuses only on the area of interest and provides more reliable results in estimating the heart rate.
3. In the previous work, the heartbeat signal’s peaks and troughs were determined from raw temporal local video signals using a peak detection algorithm with two thresholds. Here, peak and trough detection is obtained from extracted pulsatile signals, after applying a band pass filter, as in a basic PPG measurement system [23]. This approach provides more accurate detection.
4. Furthermore, an increased spatial resolution of the physiological features inserted into the classifier has the potential to capture information about micro-expressions.

## 2. Dataset Formation and Management

Experiments were conducted at Ben Gurion University in the years 2018–2020 [17]. A large dataset was created, which included short video recordings of participants’ faces passively viewing short video clips designed to evoke different emotions, two fundamentally positive and two negative emotions that are believed to be extremely distinct from each other. In line with that, the targeted emotional types in this study were disgust [D], fear [F], amusement [A], sexual arousal [S], and neutral [N] as a baseline, which were chosen from [24] that presented a wide range of emotion types beyond the standard types commonly used [25]. Participants’ faces were captured simultaneously by three cameras with different wavelength sensitivity: regular visual camera (RGB), near-infrared camera (NIR), and long-wave infrared camera (LWIR). A total of 110 subjects (63 females and 47 males) ages 18 to 33 (average age 24.6) participated in the experiment. They were then seated in front of a screen in a small room with complete privacy, with their faces spatially fixed using a special chin mount. A total of 150 different emotion-stimulating short video clips, all validated [17], with varying lengths of approximately 4–15 s (average duration 7 s) was presented to each subject, producing a total of 16,500 facial videos. The order of the played videos was set in blocks of five videos of the same emotion class (six blocks per each emotion class). The blocks were shuffled into four different sequences of block orders to be used randomly per experiment. While the emotion-eliciting videos were playing, the triple cameras were recording videos of the subject’s face at 30 frames per second. To ensure size consistency, firstly, as conducted in [17], all recorded face videos were uniformly sliced in time to include their initial 120 frames, equivalent to a 4 s duration. Videos that were just shorter than a 4 s duration were excluded. In addition to the use of validated emotion-eliciting video clips in the experiment, at the end of each played emotion-eliciting clip, a pop-up window was raised that asked the subject questions for feedback regarding the way he/she felt about the short video watched in terms of arousal and valence. It was found that these measures were correlated on average with the elicited emotion type [17]. Such validation was important due to the short length of the video clips and the passive viewing that may cause weaker emotional responses and hardly recognizable facial expressions of the subjects. In addition, emotion-eliciting videos (that participants watched) that did not demonstrate emotion-eliciting characteristics within their initial 4 s duration were also excluded from the analysis. This step ensured that the selected videos were consistent in their emotional content and relevance for the study. Overall, this process yielded a total of 130 emotion-eliciting videos per experiment for each subject (out of the original 150) for further analysis. Therefore, the 5 video classes (originally 30 video clips in each class) were now imbalanced with the following counts: sexual, 27 videos; neutral, 30 videos; disgust, 26 videos; fear, 22 videos; and amusement, 25 videos. In addition, we removed facial videos in which the subjects significantly moved their heads during the first four seconds of the experiment. Details of this operation are presented in Section 3.1.3. These imbalanced data were later handled, as explained in Section 3.3, where the classification model is described.

## 3. Method

The method is divided here into three stages: (1) finding the face region and, within it, the skin region, and, based on the skin region, estimating the heart rate and removing video records with significant head movements; (2) physiological feature extraction, which includes reshaping into low and high image sizes, from which the spatiotemporal pulsatile signal is extracted, and its peaks and troughs are detected (using the estimated heart rate), which are then used for the physiological spatiotemporal feature formation; and (3) emotion classification using a deep learning model trained by the extracted features. These stages are described in Section 3.1, Section 3.2, and Section 3.3, respectively.

### 3.1. Initial Processing and Heart Rate Estimation

#### 3.1.1. Facial ROI Cropping

**Figure 1:**
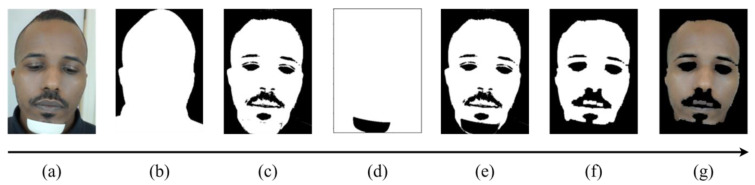
Skin segmentation process: (**a**) facial ROI frame obtained using Viola–Jones detector [26]; (**b**) semantic segmentation using MobileNetV3 [28] implemented via MediaPipe; (**c**) skin segmented via k-means algorithm (k = 2); (**d**) chin stand mask binarized with a static threshold; (**e**) multiplication of (**c**,**d**); (**f**) the final binary mask after morphological opening; (**g**) an original frame multiplied by the binary mask (skin tissue frame).

The first step in the video emotion classification process is to detect the facial region of interest (ROI). For this task, we implemented two algorithms, one for the RGB and NIR cameras and a second for the LWIR camera. For the RGB and NIR cameras, for every 10th frame, the coordinates of the face (a rectangle around the face) were located using pre-trained machine learning based on the Viola–Jones detector [26], implemented by the Open Computer Vision Library (OpenCV). Once the faces in the sampled frames were successfully detected, the coordinates of the faces were averaged, and the original video was cropped based on these new averaged coordinates (an example of an ROI for an RGB frame can be seen in Figure 1a). For the LWIR camera, an average normalized image was created from three sampled frames (uniform sampling) and blurred using a 5 × 5 averaging kernel. Then, Otsu-adaptive thresholding [27] was used to separate the face and the background (which have different temperatures); this process returned a binary image mask of the facial region. After completing this step, each frame in the LWIR camera was cropped according to the bounding box of the identified ROI.

#### 3.1.2. Skin Segmentation 

Following the facial ROI rectangular cropping stage (Figure 1a), the face video recording was segmented to distinguish the person from the background (Figure 1b) at each 10th frame (a total of 12 frames for each facial video) via a pre-trained convolutional neural network (CNN), which works as a semantic segmentation based on MobileNetV3 [28], and was implemented using MediaPipe, a cross-platform, customizable machine learning (ML) solution for live and streaming media [29]. After every frame in the sample sequence was segmented (background removed), each frame in the blue channel was binarized using the k-means algorithm (k = 2) and followed with a logical AND operation between these masks, resulting in effectively removed non-skin regions within the segmented face sequence, as depicted in Figure 1c. Additionally, about half of the video records were with a white chin stand (black in the other half), which often misled the k-means algorithm (detected as skin). Therefore, we clustered the chin stand with a static threshold of 185 (Figure 1d). Then, to obtain the desired binary mask that contained only pixels associated with the skin, we multiplied the chin stand mask with the skin, segmented via k-means (Figure 1e), and applied a morphological opening with a 10 × 10 block for producing a spatially smoother skin area, yielding the final binary mask (Figure 1f). To obtain the desired skin tissue video, we multiplied the resulting spatial mask with the original video frames (Figure 1g presents a single skin tissue frame). 

#### 3.1.3. Removing Video Records with Head Movements

To avoid head movement distortions in the facial video dataset, using the segmented skin region, we removed video records in which the subjects moved significantly during the initial 4 s of the experiment. This was conducted using the existing overlapped skin mask region (Figure 1d), which is an AND operation between detected face masks along the video. A threshold of 35% (found empirically) was applied to the ratio between the pixels that belonged to the overlapped skin mask and the total number of pixels in the image, $\frac{sum\left(mask\right)}{H*W}<0.35$. Overall, 112 videos were removed out of the 130 × 110, ending with a total of 14,188 short facial videos. 

#### 3.1.4. Estimation of Heart Rate

**Figure 2:**
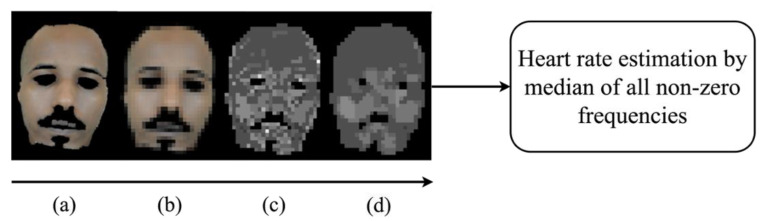
HR estimation process: (**a**) skin mask frame; (**b**) spatially down-sampled frame with averaging blocks of 10 × 10 pixels; (**c**) a spatial map of local temporal frequencies with maximum energy for the green channel (gray-level values corresponding to temporal frequencies); (**d**) smoothing the map in (**c**) using a 3 × 3 median filter. The estimated heart rate (EHR) is the median of all non-zero frequency values.

The next step was to estimate the heart rate (EHR) from the skin region for each video. The EHR was employed later in the pulsatile signal peaks and troughs detection procedure (Section 3.2.2). For this goal, the following process was applied. As in [17], firstly, spatial down-sampling with averaging blocks of 10 × 10 pixels (Figure 2b) was applied to the skin tissue video to smooth the noisiness in the temporal signals (mostly due to quantization). Then, to remove the baseline wandering and to reduce motion artifacts [23,30,31], every temporal signal (in the green channel) associated with a spatial location was bend-pass filtered using a 6th order Butterworth filter with cut-off frequencies of 0.75–3.5 Hz, corresponding to 45–210 bpm, which was the expected heart rate frequency band. Finally, FFT was applied to each one of those pulsatile signals. Using the frequency corresponding to the peak in the FFT magnitude for each spatial location, a 2D map of frequencies was generated. This map, depicted in Figure 2c, represents the spatial distribution of the highest energy temporal frequencies (a spatial distribution of the local EHR values) across the skin region. To reduce noisiness in the 2D spatial frequency map, we applied a spatial median filter with a kernel size of 3 × 3 pixels (Figure 2d). The EHR was derived from this spatial map by identifying the most common frequency value within the areas containing skin tissue only, which was calculated by the median of all non-zero elements.

### 3.2. Physiological Feature Extraction Process

#### 3.2.1. Reshaping to Fixed Image Sizes and Noisiness Reduction

Following the face detection in Section 3.1.1, the face video recordings were cropped to fit a square shape and scaled into two different resolutions, one of 56 × 56 pixels (low resolution) for obtaining improved temporal information of the pulsatile signals (due to spatial averaging which reduces the quantization noise) and one of 224 × 224 pixels for saving the spatial information of the high resolution. This resolution also fits a conventional input size of artificial neural networks (pre-trained models). To achieve a 56 × 56 pixel, low-resolution face video, first, each RGB face video frame was down-sampled with averaging blocks of 10 × 10 pixels and with 5 × 5 pixels for the NIR channel (as its original resolution was smaller). Then, a bi-cubic interpolation was performed spatially to obtain a uniform resolution of 56 × 56 pixels. Since each spatial pixel in a video frame is a part of a temporal video signal, spatial local averaging means averaging the pulsatile temporal signals of that small local region, which results in a noisiness reduction in the pulsatile signals. For the 224 × 224 pixel resolution, the process was similar; we down-sampled the RGB video records with 2 × 2 pixel blocks, and a spatial bi-cubic interpolation was performed for each image in all the cameras (RGB, NIR, and LWIR) to obtain a uniform resolution of 224 × 224 pixels.

#### 3.2.2. Pulsatile Signal Peaks and Troughs Detection

**Figure 3:**
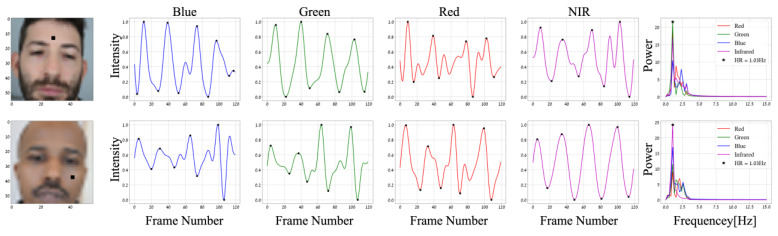
Pulsatile signal peaks and troughs. Left—black squares mark spatial locations at face images of two subjects in a 56 × 56 pixel resolution. Middle—the min-max normalized band-pass (0.75–3.5 Hz) filtered temporal signals of these locations for the B, G, R, and NIR channels, where detected peaks and troughs are marked with black dots. Right—their representation in the frequency domain, where the maximum-energy frequency is the locally estimated heart rate, marked with a black star.

The facial video frames used for the physiological feature formation are the ones time-located at the peaks and troughs of the pulsatile heartbeat signal. To obtain the time-location of these peaks and troughs, for every spatial location in the low resolution (56 × 56 pixels for RGB and NIR images), we band-pass filtered the temporal signal using a 6th order Butterworth filter with cut-off frequencies of 0.75–3.5 Hz (a similar process as in heart rate estimation in Section 3.1.4). Then, a peak detection algorithm was applied (Figure 3) with a condition of a minimal distance between neighboring samples. This distance was set to $d=\left\lfloor \frac{3}{4}\frac{T_{HR}}{T_{S}}\right\rfloor$ samples, where ⌊ ⌋ is the floor function; the period $T_{HR}$ is given by $T_{HR}=\frac{1}{f_{HR}}$ where $f_{HR}$ is the EHR frequency in Hz, which is determined in Section 3.1.4; and $T_{S}=\frac{1}{f_{s}}$ is the sampling period, where $f_{s}$ is the frame rate in Hz. This condition of minimal distance was set to be proportional to the number of samples in one period, and it is intended to avoid false positives by ensuring that detected peaks are sufficiently separated in time. In cases where lower numbers of peaks and troughs appeared during the four seconds, their arrays were zero-padded to fit the maximum length, which was set to 5 for each array (one for peaks and one for troughs). To take advantage of the high spatial resolution of 224 × 224, we up-sampled the two arrays of 56 × 56 × 5 (for the peaks and troughs) by applying the nearest-neighbor interpolation, which repeats each location 4 times for each row and column, followed by obtaining the image values at the peaks and troughs in the 224 × 224 resolution video. For each channel, this process returned two arrays of 224 × 224 × 5 of peaks and troughs ($I_{max}^{\lambda }$ and $I_{min}^{\lambda }),$ which represented 10 images at the highest and lowest energy in the video, and we call them pulsatile images. Since the LWIR (thermal) camera did not show any information related to the heartbeat as noted in [17], its output was uniformly sampled to keep only five frames used as a feature. 

Figure 3 presents two examples of band-pass filtered signals (pulsatile) at local regions, scaled (for visualization) to 0–1 range with min-max normalization, i.e., $x_{scaled}=\frac{x-min(x)\;}{\max\left(x\right)-min(x)}$ and with their peaks and troughs values marked by black dots. At the right of the figure, the corresponding frequency representation is shown (after applying the FFT algorithm), and the local EHR is marked with a black star. The two subjects (shown on the left) have different skin tones, and their pulsatile signals were obtained from two different areas. The number of peaks and troughs and the separation between them can vary between subjects. This is mainly due to differences in their HR frequencies. In addition, the beginning of the recorded PPG signal is random (during systolic or diastolic phases) and can also affect the location and number of peaks in the 4 s recording. 

The output of the peak detection algorithm is the peaks and valleys in time at every location, and the signal values in these locations (in time and space) are the pulsatile image $I_{max}^{\lambda }\left(x,y,n\right)$ and $I_{min}^{\lambda }\left(x,y,n\right)$ (Equations (4) and (5)). These images look similar to the facial video images but with slight brightness differences hardly visible to an observer.

The EHR (see Section 3.1.4) was determined from the green channel due to the following several reasons [32,33]: (1) Deoxy-hemoglobin and oxy-hemoglobin show the highest absorption spectra at the blue and green wavelengths followed by the red and NIR wavelength. (2) The signal-to-noise ratio (SNR) for green and blue wavelengths is higher than the red and NIR because of their comparatively shorter path lengths and penetration depths, making them less susceptible to noise from motion (from deeper areas such as bone and fat). (3) In the Bayer-pattern cameras, the green channel is half of the entire sensor and, hence, less signal reaches the blue and red channels, which tend to have lower SNR compared to the green channel.

#### 3.2.3. Physiological Feature Formation

Facial expressions are overall not visually observed in our emotion-elicited facial videos, which were captured when subjects viewed short emotion-eliciting video clips while sitting alone in a room without any social interaction [17,34]. A unique pre-classification feature extraction process was applied to extract possible transdermal spatiotemporal physiological changes affected by the emotional state, as suggested to occur in [22] and examined initially in [17]. Following the facial video analysis performed in at which heartbeat peaks and troughs instances of the pulsatile temporal video signal are obtained, for each facial video, we have the frames recorded at these instances. Considering this, the feature extraction process is based on the Beer–Lambert law, which defines the relationship between the attenuation of light through a skin tissue and the optical properties of that skin tissue. The light reflected from the skin can be represented by the following model [12,17,23,30]:

(1) $${\;I}^{\lambda }={I_{0}^{\lambda }(R}_{D}^{\lambda }+R_{S}^{\lambda }),$$

where $I_{0}\;$ is the incident light intensity, $R_{D}\;$ is the diffuse reflection, $R_{S}\;$ is the specular reflection, and λ is the wavelength of the incident light. The specular reflection, which is a mirror-like light reflection from the skin surface, does not contain a pulsatile signal. On the other hand, diffuse reflected light penetrates the skin surface, and it is absorbed and scattered inside the skin tissues and then reflected towards the camera sensor [12,15,23,31]. The diffuse reflectance reaching the camera can be separated into two components: non-dynamic (DC) and dynamic (AC). The DC component consists of the static contribution originating from non-varying absorbers like surrounding tissue, bone, and average blood volume. In contrast, the AC component represents variations in light absorption caused by the changes in arterial diameter when the BVP flows through, which modulates the diffused reflected light. Considering the above, Equation (1) can be expressed as

(2) $$I^{\lambda }=I_{0}^{\lambda }\left(e^{-\left(A_{DC,\lambda }+A_{pulsatile,\lambda }\right)}+R_{S}^{\lambda }\right)$$

(3) $$I^{\lambda }={I_{0}^{\lambda }(R}_{DC}^{\lambda }R_{pulsatile}^{\lambda }+R_{S}^{\lambda }),$$

where $A_{DC,\lambda }$ and $A_{pulsatile,\lambda }$ denote the absorbance of the DC and AC components, respectively, which depend on the absorption coefficients, optical path length, and concentration of the medium. $R_{DC}^{\lambda }$ is the total DC reflection from the static contribution absorbers, and $R_{pulsatile}^{\lambda }$ is the total reflection caused by changes in arterial blood volume pulse.

Drawing upon the research conducted in the domain of contact PPG applications for clinical environments, a comprehensive set of features associated with the pulsatile signal and hemoglobin concentration changes has been developed [11,12,16,23,35]. In the context of the present study, these well-established features have been harnessed and used in a cbPPG to classify personal emotion states as examined in [17]. This methodology does not only utilize temporal information but also extends to spatial dimensions; i.e., the temporal pulsatile signals at the different facial skin locations. Based on the signal’s peaks and troughs indexes, images at these time instances are formed (Section 3.2.2). These images, $I_{max}^{\lambda }\left(x,y,n\right)$ and $I_{min}^{\lambda }\left(x,y,n\right)$, indicate the physiological characteristics (see the explanations of Equations (4) and (5), where x and y denote the spatial locations, and n denotes the time index along the facial pulsatile video).

- Maximum signal amplitude (at the peaks): pixel’s gray level during the diastolic phase for wavelength λ (arterial diameters are minimized; thus, absorbance is minimized while the amount of light detected is maximized) => $A_{pulsatile,\lambda }^{min}=>I_{max}^{\lambda }\left(x,y,n\right)$.
  (4) $${{I_{max}^{\lambda }\propto I}_{0}^{\lambda }(R}_{D}^{\lambda ,max}+R_{S}^{\lambda }).$$

- Minimum signal amplitude (at the troughs): pixel’s gray level during the systolic phase for wavelength λ (arterial diameters are maximized; thus, absorbance is maximized while the amount of light detected is minimized) => $A_{pulsatile,\lambda }^{max}=>I_{min}^{\lambda }\left(x,y,n\right)$.
  (5) $$I_{min}^{\lambda }\propto {I_{0}^{\lambda }(R}_{D}^{\lambda ,min}+R_{S}^{\lambda }).$$
  Based on the above pulsatile images, the following features, found to be the most effective for emotion classification, were used in this study.

- The amplitude of the pulsatile signal is
  (6) $${I_{AC}^{\lambda }\left(x,y,n\right)=I}_{max}^{\lambda }\left(x,y,n\right)-I_{min}^{\lambda }\left(x,y,n\right),$$
  where $I_{AC}^{\lambda }\propto {I_{0}^{\lambda }(R}_{D}^{\lambda ,max}-R_{D}^{\lambda ,min})={I_{0}^{\lambda }R}_{DC}^{\lambda }(R_{pulsatile}^{\lambda ,max}-R_{pulsatile}^{\lambda ,min}).$

- The absorption amplitude that eliminates the effect of static absorbers is
  (7) $${\;I}_{R}^{\lambda }\left(x,y,n\right)=ln\left(\frac{I_{max}^{\lambda }\left(x,y,n\right)}{I_{min}^{\lambda }\left(x,y,n\right)}\right),$$
  where $I_{R}^{\lambda }\propto \ln\left(\frac{R_{D}^{\lambda ,max}+R_{S}^{\lambda }}{R_{D}^{\lambda ,min}+R_{S}^{\lambda }}\right).$

Each spatial-temporal feature is eventually of size 224 × 224 × 5, as explained in Section 3.2.2.

### 3.3. Emotion Classification Models

Due to a few data management operations detailed in Section 2 and Section 3.1.3, some imbalances appeared in the final dataset with respect to the categories (i.e., emotion classes). Overall, these processes yielded a total of 130 × 110–112 face videos (out of the original 150 × 110 recorded), which are not perfectly balanced in the number of videos per subject. To mitigate the effects of the imbalanced dataset without modifying the minority and majority class ratio by performing over/under sampling methods, we instead used the weighted loss method, at which higher weights are assigned to the smaller classes. This gives a higher importance to the smaller class samples during training and reduces the bias towards the larger class.

For emotion classification using the physiological features, we considered various deep learning approaches, which can be divided into two branches. The first includes models that were built upon CNN principles, and the second includes models based on vision-transformer (ViT) principles. We also examined training models from scratch versus transfer learning by fine-tuned, pre-trained models. The highest-accuracy classifier was a pre-trained EfficientNet-B0 [36], while a pre-trained Swin-Transformer [37] achieved the second-best results (with different input features). Considering this, a pre-trained EfficientNet-B0 model was applied in this study to classify individual emotional states based on physiological features that were created from pulsatile images. EfficientNet is an architectural and scaling technique for CNNs that uses a compound coefficient to scale all depth, width, and resolution dimensions in a balanced manner. The architecture of the EfficientNet-B0 uses a 224 × 224 × 3 input dimension with 16 mobile-inverted bottleneck convolutions (MBConv), with kernel sizes of 3 × 3 or 5 × 5. Each MBConv contains a squeeze and excitation (SE) block, batch normalization, SiLU activation function, depthwise convolution, and pointwise convolution which are characterized by a relatively low number of parameters (which reduces the training time), as compared to the other state-of-the-art models [36]. To ensure the robustness of our findings and increase the utility of the extensive but limited experimental dataset of 110 subjects, a cross-validation approach was employed for training and testing. Specifically, a “leave-5-subjects-out-cross-validation” [38] technique (i.e., 22 non-overlapping subsets of 5 subjects in a 22-fold cross-validation( was adopted for validation. In addition, all the model weights were initialized using pre-trained models trained on the large imagenet-1 K dataset, available in the Torchvision library, which is an integral part of the PyTorch project [39]. 

**Figure 4:**
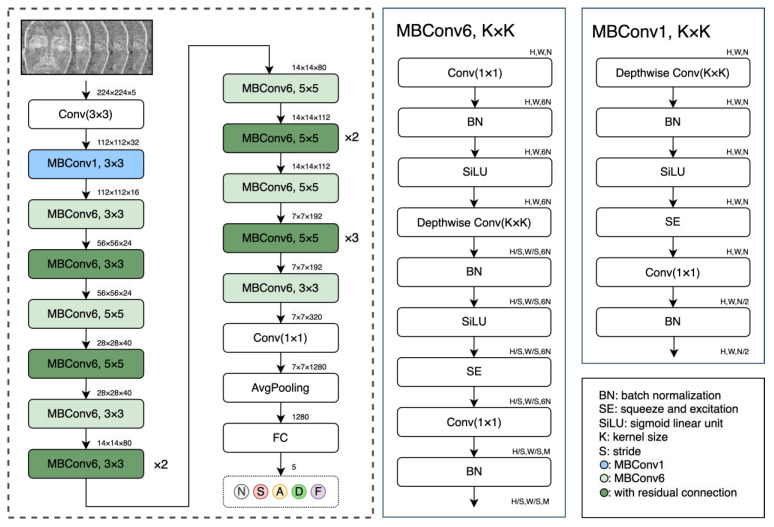
The EffecientNet-B0 general architecture (right), with the building blocks MBConv1 and MBConv6 (detailed at the left). The input is a 224 × 224 × 5 block that contains five pulsatile feature maps (for a four-second facial video), classified into five emotional states.

To perform transfer learning, we made modifications to the first and last layers of our models. The pre-trained model requires an input size of 224 × 224 × 3 (height × width × number of channels); however, our physiological features have a size of 224 × 224 × 5 (height × width × features length). To accommodate these different input sizes, we adapted the input layer of our backbone model accordingly. Additionally, the last layer of the model was also modified due to the number of classes in our dataset, which corresponds to five emotional states (Figure 4). The following hyper-parameters have been used to train the EfficientNet-B0 model: iterations, 25; learning rate, 0.001; batch size, 64; and an increased dropout of 0.5 for a higher regularization. Data augmentation was also applied to the training process for increasing the limited data samples, including random rotations of 90, 180, and 270 degrees and randomly closing a pixel (with a uniform probability of 1/124) in the time domain by setting the corresponding temporal signal at that pixel to 0. 

Furthermore, we trained the examined models with a variety of input feature combinations (with single wavelengths and combinations of different wavelengths) constructed from the pulsatile images (see Equations (4) and (5)). In [17], eight features are presented, and all of them were used together as an input to a CatBoost classifier. Here we found that single input features with a single wavelength (see Equations (6) and (7)), obtained from the RGB camera, can produce the best accuracies on our dataset when DL models are employed, which were higher than the results obtained in [17]. Note, however, that a single feature includes very rich, subtle data of 224 × 224 × 5 values, as explained in Section 3.2.3.

**Figure 5:**
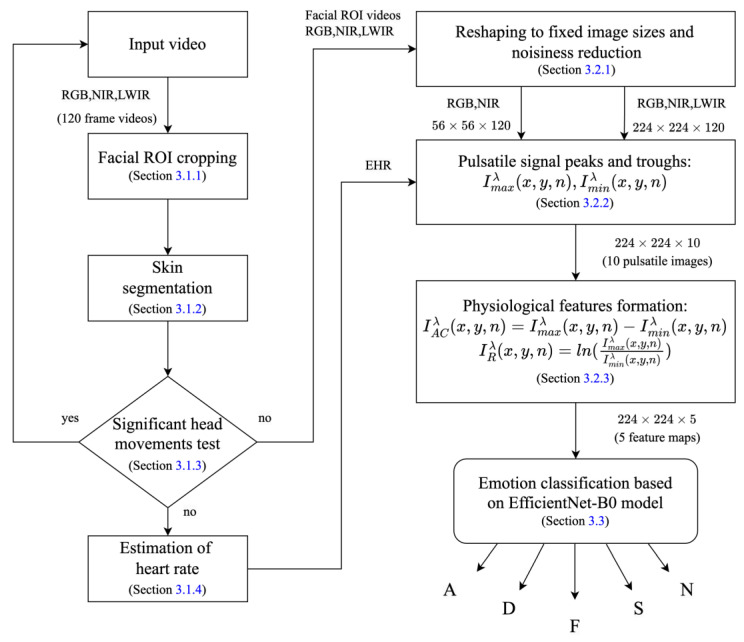
A block diagram of the proposed algorithm for emotion classification. The outputs A, D, F, S, and N represent the emotion classes: amusement, disgust, fear, sexual arousal and neutral, respectively.

A schematic diagram of the proposed method is presented in Figure 5. The inputs are the RGB, NIR, and LWIR video frames of the emotion-stimulated subjects’ faces.

## 4. Results

**Figure 6:**
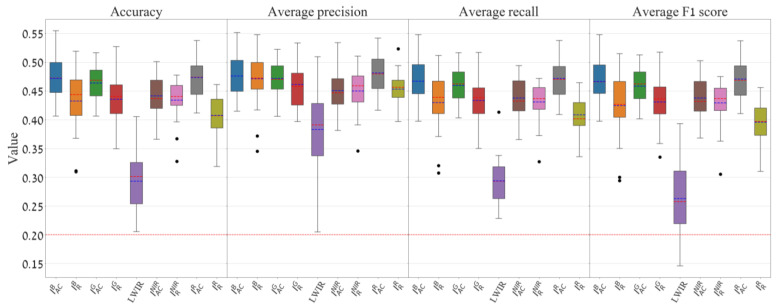
Average measurements of the models for nine different input features according to the overall accuracy, average precision, average recall, and average F1 score per feature. The red dashed horizontal line represents the random classifier for five classes (20%). Values outside the 1.5 IQR range are marked with black dots. The red and blue dashed lines inside the IQR rectangles denote the median and average, respectively. The nine different box colors represent the nine different input features shown at the horizontal axis.

**Figure 7:**
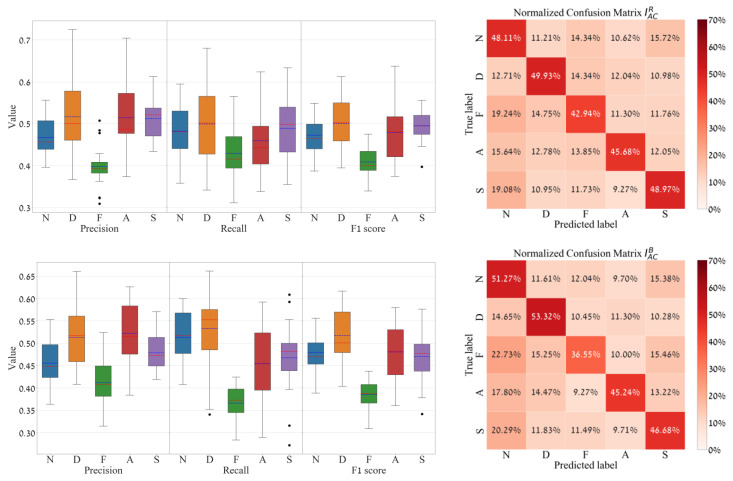
Multiclass classification results for features $I_{AC}^{R}\;$(**Top**) and $I_{AC}^{B}\;$(**Bottom**), which represent the pulsatile amplitudes of the red and blue wavelength channels. Right: the row-normalized outcome of the sum of all the resulting 22 model folds’ confusion matrices. Left: model’s statistical performances per emotion class, according to the precision, recall, and F1 score. The five different box colors represent the five emotion classes (N, D, F, A and S) marked at the horizontal axes.

The common evaluation metrics for multi-class classification per class (i.e., micro measurement) are precision, recall, and F1 score. Also, a global measurement can be applied, as it is determined by averaging the individual measurements per class (i.e., macro measurement), and they are averaged by precision, averaged recall, averaged F1 score, and accuracy (the exact match) [40]. To evaluate the models in the leave-5-subjects-out-cross-validation, the following three metrics were used: (a) global measurements per model, which include accuracy (exact match), average precision, average recall, and average F1 score (all are summarized through feature-based boxplots (Figure 6)). (b) A row-normalized confusion matrix was created from the sum of all the resulting 22 model folds’ confusion matrices per unique feature (Figure 7, right). (c) For every confusion matrix generated from the 22 models, three statistical assessments were applied per emotion class with precision, recall, and F1 score, and the comprehensive results across all 22 models are succinctly summarized through a class-based boxplot (Figure 7, left).

The classification outcomes for nine different input features are summarized and presented in Figure 6. The features that produced the highest accuracy are $I_{AC}^{R}$ (M = 47.36%, SD = 3.47%) and $I_{AC}^{B}$ (M = 47.15%, SD = 3.63%) using the EfficientNet-B0 model, where M and SD denote the mean and standard deviation, respectively. In addition, the results highlight the weaker performance of the LWIR feature with M = 0.29 and SD = 0.04. Features based on the NIR channel produced somewhat weaker performances than the regular RGB channels.

Furthermore, per-emotion class evaluations of the classification performances are depicted in Figure 7 (using the best input features, $I_{AC}^{R}\;and\;I_{AC}^{B}$), as a row-normalized confusion matrix (sum over all the 22 confusion matrices and then row-normalized) and alongside a boxplot measurement (precision, recall, and F1 score (per emotion class. According to this measurement, for $I_{AC}^{R},$ the results indicate that the disgust (D) class was precisely identified 49.93% of the time, followed by the sexual arousal class (S) with 48.97%, the neutral (N) with 48.11%, the amusement (A) with 45.68%, and, lastly, fear (F) with 42.94%. In addition, for feature $I_{AC,}^{B}$ it was a little different, as the sexual arousal class changed place with neutral. It can also be seen that the fear class achieved the worst prediction precision in both features (42.94% for $I_{AC}^{R}$ and for $I_{AC}^{B}$ is 36.55%).

## 5. Conclusions

In this study, we improved a new emotion classification approach that does not rely on stereotypical facial expressions or measurement equipment attached to the body. The features examined for the classification were extracted from facial video recorded remotely by three cameras (RGB, NIR, and thermal). The features that were used as inputs to the classification system were calculated from pulsatile-based heartbeat facial video frames that may represent spatiotemporal physiological characteristics affected by emotions. In this study, we have found that single unique input features extracted from RGB facial videos can produce better emotion classification when using an appropriate classifier. These features can be the difference or the division between frames at following peaks and troughs of the pulsatile video. The pulsatile frames are of a single visual color component (wavelength region), such as red or blue, and are calculated at the peaks and troughs time locations in the video by using the EHR, which is estimated from the segmented skin. In this way, we obtained 47.36% average classification accuracy using a single feature ($I_{AC}^{R}$) and a DL classifier, compared to the previous work of [17] that used a bulk of eight features and a CatBoost classifier, which obtained 44% accuracy. This indicates that a regular RGB camera, which is commonly available and relatively inexpensive, can produce promising emotion classification results. In addition, it was found, as in [17], that for short video signals, the thermal camera was the worst among the tested cameras. It is important to note, however, that the performances of the features for indicating emotions may depend on the length of the emotion stimulation and also on the sensitivity and sensor quality of the cameras. 

The limited classification accuracy obtained in this study stems from the challenging nature of the dataset for the following reasons: (1) The short length (four seconds) of the facial videos of the subjects that viewed emotion-eliciting videos may limit the behavioral reactions in the observers, as part of the emotion-eliciting videos may not immediately elicit a significant emotional response. Additionally, a significant thermal response usually starts more than ten seconds after the stimulus onset [41]. (2) In the experiment, it was assumed that emotional stimuli automatically elicit behavioral reactions in the observer, in line with the motivational theories of emotions [42,43]. However, as a series of very recent studies showed, emotional facial expressions elicited a consistent, replicable behavioral effect only when they were relevant to the participants’ goals [44,45]. This would mean that passive viewing may not be the best way to induce an emotional response. 

Compared to other methods, as stated in this paper’s introduction, facial expression recognition methods that can reach classification success rates surpassing 90% are the most popular and studied non-contact approaches for emotion classification [3,4]. But facial expressions may also correspond to communicative intentions and can be manipulated to deceive and, thus, may not necessarily express true emotions. Furthermore, facial expressions are known to be interpreted very well by humans. For example, reference [46] found that human observers performed better than all eight commercially available automatic facial expression classifiers examined. In our dataset, however, human observers obtained only an average of about 30% classification accuracy (compared to 20% chance) [34], which is clearly lower than our 47.4% accuracy. This means that our approach, while being non-contact, performs a more challenging task and has the potential to extract and classify uniquely delicate facial physiological signals due to lower-intensity and less-expressed emotions, which other methods may not be able to detect.

It is important to note that, while our method relies primarily on pulsatile image signals since we removed 112 videos from the dataset due to head movements and used a relatively high spatial resolution, minor motions/micro-expressions, if they exist, may affect the classification. The method, while promising, faces certain practical limitations in the current setup. Firstly, despite being a contactless approach, it requires subjects to maintain a reasonable distance from the camera. Secondly, it requires the subjects not to move during the imaging for effective operation when no image registration is applied. In order to allow head movements and combine them in the automatic classification procedure, a process of face tracking and registration can be applied (following the face detection stage presented in Section 3.1.1). This can cause the method to be more applicable in real-world situations.

## Data Availability

The datasets presented in this article are not readily available, because they include facial videos of subjects without consent for public distribution yet. Requests to access the datasets should be directed to S.T. or Y.Y.
